# Annotations

**Published:** 1896-10-17

**Authors:** 


### Annotations.

### Teaching in Medical Schools.

In the introductory address which he gave at the Sheffield Medical School, Sir Henry Littlejohn described the teaching arrangements of the extra-mural school at Edinburgh, which has now no fewer than sixty-five lecturers attached to it, and has become the largest school of this kind in the world. How, then, does this most successful school supply itself with teachers? How is it that while students at other schools suffer from the dulness of their lecturers, this school can supply not only itself but other educational centres with professors? First of all by never insisting that those who wish to join as teachers shall have been previous students of the school; and next, by leaving admission to the teaching staff open to all and sundry who may choose to apply, and allowing anyone to select what subject he pleases for his lectures. An applicant must fulfil certain conditions, but if these requirements are satisfactorily met he is entitled to teach for university graduation and for the diploma of the college. "It matters not how many lecturers there may be already engaged on the subject he may choose. The French apophthegm holds true, *La carrière ouverte aux talents*. The new lecturer soon finds his level; the student quickly discovers where he can get the most value for his money, and a lecturer who fails to attract students by the excellence of his teaching soon goes to the wall." How many London teachers would not go to the wall under such a system of open competition! It is a matter worthy of very serious consideration whether the London schools could not vastly increase their teaching capacity by the introduction of some analogous system, allowing of much more open competition in this matter than exists at present. Even a system of co-operation and interchange between the different schools would be a great step in advance. In its opportunities for clinical study London is unequalled, but he would, indeed, be an optimist who would maintain that the lectures offered to its students could not be improved. Yet there are lecturers in London who are not only good, but brilliant. Unfortunately to whichever school the student attaches himself he finds that the brilliancy of one teacher is tempered, if not neutralised, by the unutterable dulness of another. Even the post-graduate courses have not been able to escape from the rut. Yet by a little co-operation, a little breaking down of the isolation of the different schools, what a galaxy of distinguished teachers might be available for London students.

### Doctors and the Peerage.

Sir William O. Priestley, M.P., who is well-known to most readers as a distinguished M.D., repeated at University College, Liverpool, a very ancient protest on the part of the medical profession in general. He told the assembled students that whilst soldiers, lawyers, and clergymen constantly received the highest honours which our British State can confer, and even poets and artists have of late years shared in such distinctions, the medical profession, notwithstanding all it has recently done and is now doing for the advancement of science, and the saving of life and health, is still almost as much neglected by both political parties as it was in the worst days of the pre-scientific period. Sir William thinks this is a foolish failure on the part of the State to give recognition to a class of persons who serve it at least as well as, and in many respects better than, most other classes—and so think we. But if the medical profession is to be raised in this regard, it will have to help itself. The only effective way in which this can be done is by the acquisition of political influence. If the medical profession could become definitely political, like the legal and the clerical, it would soon, like them, have its peerages. In the meantime, since we are all too busy striving to lay by fortunes greater or smaller, there seems to be very little prospect of any general effort in this direction.

### The Lack of Decency in Factories.

Buried in the report of H.M. Chief Inspector of Factories, a volume which is probably not so widely read as it deserves to be, are certain observations of the female inspectors in regard to the sanitary arrangements of factory life. These ladies are much to be commended for so prominently bringing forward the moral aspect of what, for the sake of euphemy, we may call the lavatory question, although the name is not quite appropriate, for we fear that in too many cases wash-basins are quite a dream of the future. People who are accustomed to look upon foreigners, taking them "in the lump," as greatly inferior to ourselves, especially in matters of virtue and propriety, will be shocked to hear that "England appears to be the only great industrial country in Europe which has no clause in its factory legislation requiring general propriety as well as health in these arrangements." Miss Paterson, speaking of sanitary accommodation for women and children in the Lancashire mills, says: "Defective in structure, in light, in cleanliness, in ventilation, as well as objectionable in situation, the conveniences provided are, from the point of view, both of health and decency, deplorable. . . . . In those factories, especially in which a large number of children are employed, the moral injury caused by the present system can scarcely be over-estimated . . . and it is a serious matter to place them amid surroundings which may, and indeed must, tend to blunt their sense of modesty." It appears that too often it is the case that the sanitary conveniences for men and women are placed alongside of each other with utter disregard of privacy, while in some cases the accommodation for women is placed in a part of the factory where men only are employed. In one case, "not only were the conveniences opening directly into the work-rooms, without any kind of system of flushing or trapping, and common to both sexes, but their doors even had been removed on the ground that the workers wasted their time." It is also stated that in Rochdale, Burnley, Wigan, and other large towns in Lancashire, "such cases as I have cited are by no means isolated." These are disagreeable subjects, and all praise is due to the lady inspectors for rooting out these abominations. We can well understand one of these inspectors speaking of "the eagerness with which the women have received me, as a woman," and we can well believe that "long continued negligence in this matter has produced the inevitable consequence of a low standard of tone and behaviour." Anyone who reads these reports will quickly see that the "women inspectors" are doing a good work. One of them says: "I am told by employers that they hear no complaints from the women in their employment. They are not likely to do so; but I am resolved that they shall hear many from me," and we wish her well in her endeavour.

